# COL8A1 Predicts the Clinical Prognosis of Gastric Cancer and Is Related to Epithelial-Mesenchymal Transition

**DOI:** 10.1155/2022/7567447

**Published:** 2022-06-21

**Authors:** Yali She, Xiaowen Zhao, Pingfan Wu, Ling Xue, Shengfang Wan, Lei Zhang, Changtian Li, Hui Cai, Yaling Li

**Affiliations:** ^1^Department of Pathology, School of Basic Medicine, Gansu University of Chinese Medicine, Lanzhou, Gansu, China; ^2^Provincial-Level Key Laboratory of Molecular Medicine of Major Diseases and Study on Prevention and Treatment of Traditional Chinese Medicine, Gansu University of Chinese Medicine, Lanzhou, Gansu, China; ^3^Department of Pathology, Chinese People's Liberation Army Joint Logistics Support Unit 940 Hospital, Lanzhou, Gansu, China; ^4^Laboratory of Preclinical Medicine of the 940th Hospital of Joint Logistics Support Force of Chinese People's Liberation Army, Key Laboratory of Stem Cells and Gene Drug of Gansu Province, Lanzhou, Gansu, China; ^5^Key Laboratory of Molecular Diagnostics and Precision Medicine for Surgical Oncology in Gansu Province, Gansu Provincial Hospital, Gansu 730000, China; ^6^NHC Key Laboratory of Diagnosis and Therapy of Gastrointestinal Tumor, Gansu Provincial Hospital, Lanzhou 730000, China

## Abstract

**Background:**

Gastric cancer (GC) is the fifth most common malignant tumor and the third leading cause of cancer-related deaths. Because GC has the characteristics of high heterogeneity, unclear mechanism, limited treatment methods, and low five-year survival rate, it is necessary to find the prognostic biomarkers of GC and explore the mechanism of GC.

**Methods:**

We first identified differentially expressed genes (DEGs) between gastric cancer and normal gastric cells through expression analysis. A protein-protein interaction (PPI) network was constructed to find tightly connected modules. We performed survival analysis on the DEGs in the modules to identify genes with prognostic significance. Gene set enrichment analysis (GSEA) was used to identify gene enrichment pathways. Finally, we used our own collected clinical samples of 119 gastric adenocarcinoma (STAD) tissues and 40 normal gastric tissues to perform immunohistochemical (IHC) staining to verify the differential expression of COL8A1 in STAD tissues and normal gastric tissues and its correlation with epithelial-mesenchymal transition- (EMT-) related factors.

**Results:**

We identified 356 DEGs through differential expression analysis. Through PPI analysis and survival analysis, we determined that the collagen type VII alpha-1 chain (COL8A1) gene has prognostic significance. GSEA analysis showed that COL8A1 was significantly enriched in the EMT. IHC results showed that COL8A1 was upregulated in STAD tissues and could be used as an independent prognostic factor and was related to EMT.

**Conclusion:**

This study shows that COL8A1 is related to the prognosis of GC patients and might affect the progress of GC through the EMT pathway. Therefore, COL8A1 may be a biomarker for predicting the prognosis of GC.

## 1. Introduction

Worldwide, gastric cancer (GC) is the fifth most common malignant tumor and the third leading cause of cancer-related deaths [1]. Although progress has been made in surgery, chemotherapy, targeted therapy, and immunotherapy, the 5-year overall survival (OS) rate of GC is only 20% due to the lack of sensitive and specific biomarkers and the advanced stage at diagnosis [2, 3]. Stomach adenocarcinoma (STAD) is the main type of gastric cancer [4]. Therefore, it is extremely important to explore the mechanism of occurrence and development of gastric cancer and seek new potential biomarkers for early diagnosis and prognostic evaluation.

Type VIII collagen was originally identified as a biosynthesis product of bovine aorta and rabbit corneal endothelial cells. Collagen type VIII alpha-1 chain (COL8A1) is responsible for encoding the type VIII collagen *α*1 chain and plays a role in the proliferation and migration of different cells [5]. COL8A1 has been implicated in vascular injury, angiogenesis, and protumorigenic processes. COL8A1 is involved in the angiogenesis of certain brain tumors [6]. Silencing of COL8A1 significantly inhibited the proliferation and invasion of hepatocellular carcinoma cell lines and increased the sensitivity to D-limonene in the treatment of hepatocellular carcinoma [7]. Vastatin, a fragment of collagen type VIII, is increased in the serum of colorectal cancer patients and is associated with stromal responses [8]. However, the role of COL8A1 in gastric cancer remains unclear.

Epithelial-mesenchymal transition (EMT) is the process by which epithelial cells transform into cells with a mesenchymal phenotype, which is associated with tumor invasion and metastasis [9]. EMT contributes to the transition of gastric cancer from early to mid-late stage because it influences the aggressiveness of gastric cancer cells [10]. A variety of factors can affect the EMT process of the tumor either directly or through crosstalk. For example, multiple intracellular signaling pathways coordinate to induce EMT, and various factors secreted by cells in the tumor microenvironment can induce EMT [11]. Upregulation of vimentin and downregulation of E-cadherin are hallmarks of EMT [12, 13].

In recent years, bioinformatics has brought a turning point in tumor research. It facilitates the collection and organization of tumor research results from different perspectives and allows investigators to build various databases with different functions according to different needs [14]. The Gene Expression Omnibus (GEO) and The Cancer Genome Atlas (TCGA) are commonly used databases in cancer research. There have been many studies using bioinformatics to analyze gene expression and clinical characteristics to identify molecular biomarkers, predict prognosis, or predict drug resistance [15–17].

In this study, we downloaded four STAD-related gene sets from GEO and sequencing data from TCGA. Differentially expressed genes (DEGs) were identified by R software. Then, Gene Ontology (GO), Kyoto Encyclopedia of Genes and Genomes (KEGG) pathway enrichment analysis, and protein-protein interaction (PPI) network analyses were performed. Finally, we identified the COL8A1 gene as having prognostic significance through survival analysis. We evaluated COL8A1 enrichment in the EMT pathway through gene set enrichment analysis (GSEA). To clarify the prognostic significance and possible carcinogenic mechanism of COL8A1 in GC, we used immunohistochemistry and tissue chip technology to verify the prognostic significance of COL8A1 and its relationship with EMT.

## 2. Materials and Methods

### 2.1. Microarray Data Source

By searching in the GEO database (http://www.ncbi.nlm.nih.gov/geo/), gene microarray data (GSE19826, GSE66229, GSE79973, and GSE118916) were obtained. The inclusion criteria for these datasets were as follows: (1) They all included normal (nontumor)/tumor-matched human gastric tissue. (2) Each dataset contained at least 20 samples. In addition, we also downloaded raw RNA-sequencing data including 375 GC samples and 32 matched normal samples from TCGA (https://cancergenome.nih.gov/).

### 2.2. Data Processing of Microarray Datasets

In the R software, the “limma” package is used for screening for DEG [18]. The “RobustRankAggreg” package was used to identify common differentially expressed genes in four datasets. Since this RRA method is based on the null hypothesis of irrelevant input, its screening results are improved over prior methods [19]. The select criteria for DEG were |log2foldchange| ≥ 1 and *P* value < 0.05.

### 2.3. Validation of DEGs

The RNA-sequencing data obtained by TCGA was used to verify the results of the GEO dataset integration analysis. A |log2foldchange| ≥ 1; *P* value < 0.05 was considered statistically significant. We retained the overlapping genes of DEG obtained from TCGA RNA-sequencing data analysis and GEO integration analysis for further analysis.

### 2.4. GO and KEGG Enrichment Analyses of DEGs

We used the “clusterProfiler” package in R software to perform GO and KEGG analyses on overlapping DEGs and generated a visual analysis output of cellular components (CC), biological processes (BP), molecular functions (MF), and pathways among the overlapping DEGs.

### 2.5. PPI Analysis

STRING (https://string-db.org/) is a database for exploring known and predicted protein-protein interactions. To evaluate the interaction between these DEGs, we mapped DEGs to STRINGs and selected PPIs with confidence scores ≥ 0.7 to be retained and further imported into Cytoscape software. We used the cytoHubba application in Cytoscape software to build a PPI network. The Cytoscape Molecular Complex Detection (MCODE) plug-in was used to select the most closely connected module from the existing PPI network, and we set the filter conditions as degree cutoff = 2 to carry out further functional analysis [20].

### 2.6. Survival Analysis

The clinical information on 326 GC patients was used for survival analysis. We used the “survival” package to analyze the clinical information of these patients to find genes that were closely related to survival.

### 2.7. Gene Set Enrichment Analysis

Based on the selected candidate genes, we performed GSEA analysis using GSEA software to determine the potential function of the candidate genes. The enrichment score indicated the degree of enrichment of genes in the pathway. The annotated h.all.v6.2.entrez.gmt gene set in the Molecular Signature Database was used as the reference gene set. FDR < 0.25 was defined as the critical value.

### 2.8. Collection Patient Tissue Specimens and Clinical Information

In order to verify the differential expression of COL8A1 in gastric cancer and normal gastric tissues and the relationship between COL8A1 and EMT, we collected relevant cases for experimental verification. The included cases were 119 STAD tissue samples from 119 patients admitted to Wuwei Tumor Hospital in Gansu Province, China, and pathologically diagnosed with STAD from December 2012 to November 2020, and paired normal gastric tissue from 40 of these patients. Due to the small size of the resected tumor in some patients, there was no matching tissue available. The follow-up period was August 2021. Because the patients were anonymous, this study was exempt from signed informed consent.

### 2.9. Preparation of Tissue Microarray

Formalin-fixed and paraffin-embedded (FFPE) blocks and corresponding hematoxylin and eosin (H&E) sections of all cases were examined by two experienced pathologists. Two pathologists reevaluated the STAD specimens based on the 2010 Eighth Edition of the American Joint Committee on Cancer (AJCC) Tumor, Lymph Node, and Metastasis (TNM) classification. In addition, the pathologist circled the STAD and normal gastric tissue areas on the H&E slice of the corresponding FFPE block.

### 2.10. Immunohistochemistry (IHC)

Based on the circled area of the H&E slice, we took the tumor core from the corresponding FFPE block and transferred it to the blank FFPE block to construct a tissue microarray. Then, 4 *μ*m sections were used for immunohistochemistry.

The sections were dewaxed in xylene and hydrated via treatment with increasingly dilute ethanol solutions. The sections then were placed in ethylenediaminetetraacetic acid (EDTA) stock solution (1 : 50 dilution, pH = 9.0) for high temperature and pressure antigen retrieval. The sections were incubated with a peroxidase blocking agent for 10 minutes to block endogenous peroxidase activity and blocked with normal unimmunized animal serum according to the manufacturer's protocol. Anti-COL8A1 antibody was diluted 1 : 100 after which the sections were incubated with an anti-COL8A1 antibody (Cloud-Clone Technology Co., Ltd., Wuhan, China, catalogue number: RAC146Mu01), anti-E-cadherin antibody, and anti-vimentin antibody at 4°C for 11 hours.

### 2.11. Evaluation of COL8A1, E-Cadherin, and Vimentin Expression

The expression of COL8A1, E-cadherin, and vimentin was evaluated by two experienced pathologists. COL8A1, E-cadherin, and vimentin were all positively expressed as brown particles on the cell membrane and/or cytoplasm. Using semiquantitative results to judge the expression of COL8A1, the percentage of positive cells and staining intensity was determined under a microscope. The staining intensity was scored from 0 to 3 points (0, negative; 1, weak; 2, medium; and 3, strong). Each tumor was then scored for the percentage of immunoreactive cells. Finally, the two scores were multiplied to assign a grade: 0 is negative (-), 1-4 are weakly positive (+), 5-8 are positive (++), and 9-12 are divided into strong positive (+++). E-cadherin is positively expressed as brown particles on the cell membrane and/or cytoplasm. The E-cadherin levels were divided into positive expression (positive cells ≥ 90%) and negative expression (positive cells < 10%). Vimentin's interpretation standard is that positive cells ≥ 20% are positive.

### 2.12. Statistical Analysis

SPSS software was used to perform statistical analysis (version 26.0, SPSS Inc., IBM Chicago, IL, USA). The statistical analysis methods used include the chi-square test, rank sum test, and Spearman's rho (depending on the situation). A *P* < 0.05 was considered statistically significant.

## 3. Results

### 3.1. Identification of DEGs

Detailed information on the four GEO datasets included in our study is shown in Table 1. After a comprehensive analysis of the four datasets, a total of 528 DEGs were obtained, including 195 upregulated genes and 333 downregulated genes. A total of 11,844 DEGs were obtained from the analysis of the TCGA GC dataset, including 7879 upregulated genes and 3965 downregulated genes. The intersection analysis of the integrated microarray and RNA-sequencing results allowed us to identify 356 overlapping DEGs including 133 upregulated genes and 223 downregulated genes.

### 3.2. GO and KEGG Enrichment Analyses

**Figure 1:**
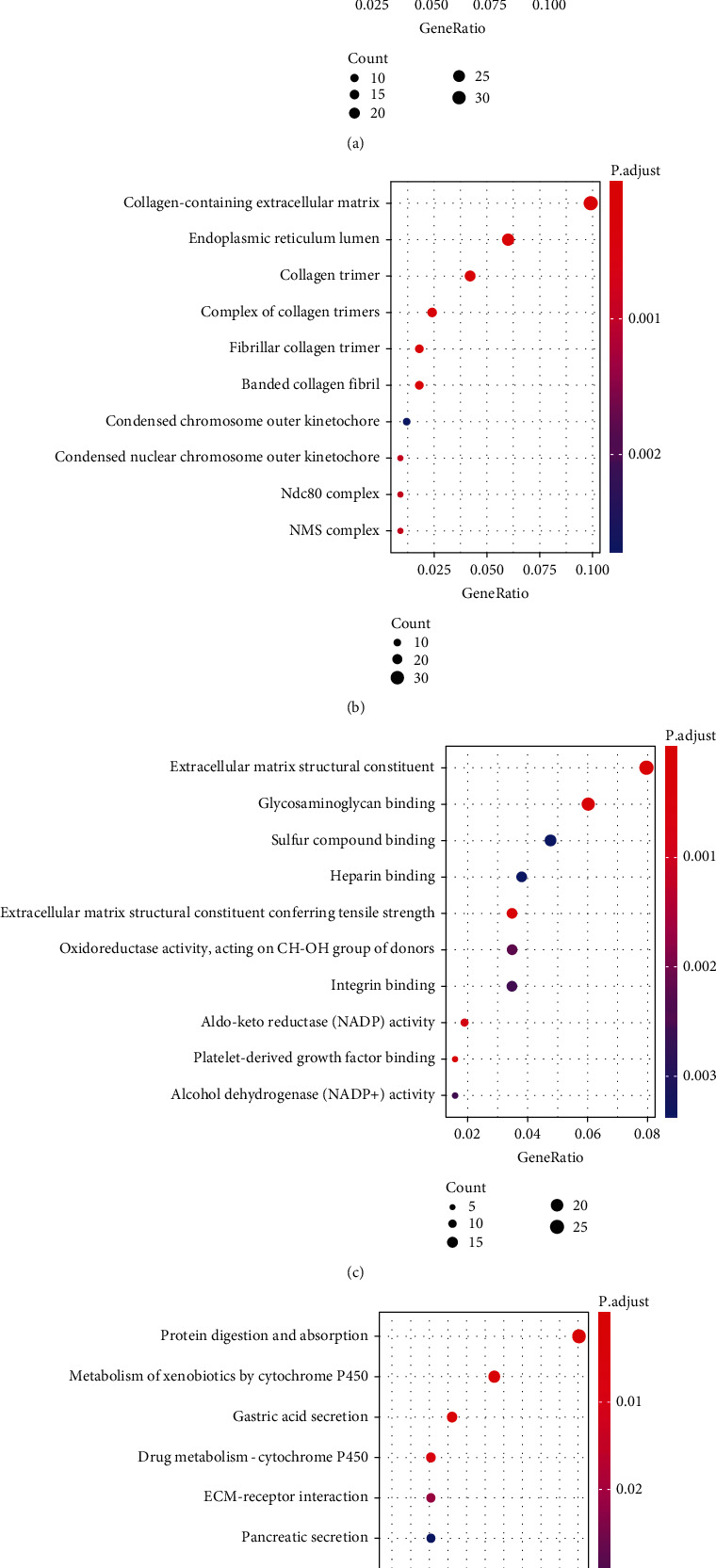
(a) Biological processes (BP) in GO analysis of overlapping DEGs. (b) Cell components (CC) in GO analysis of overlapping DEGs. (c) Molecular function (MF) in GO analysis of overlapping DEGs. (d) KEGG pathway analysis of overlapping DEGs.

We performed an enrichment analysis of GO and KEGG pathways on the 356 overlapping DEGs to determine the GO category and KEGG pathway. DEG is closely related to the collagen-rich extracellular matrix in the classification of CC (Figure 1(a)) and BP (Figure 1(b)). In terms of MF classification, DEGs were significantly enriched in the extracellular matrix structural constituent and extracellular matrix structural constituent conferring tensile strength (Figure 1(c)). These results indicate that DEGs are closely related to collagen components in the extracellular matrix, and studies have shown that collagen itself participates in many aspects of tumor transformation [21]. The results of the KEGG pathway analysis indicate that DEGs were significantly enriched in protein digestion, absorption, and metabolism of xenobiotics by cytochrome P450 (Figure 1(d)). The above results show that these DEGs were enriched in the pathways involved in the occurrence and development of GC [22, 23].

### 3.3. PPI Analysis

**Figure 2:**
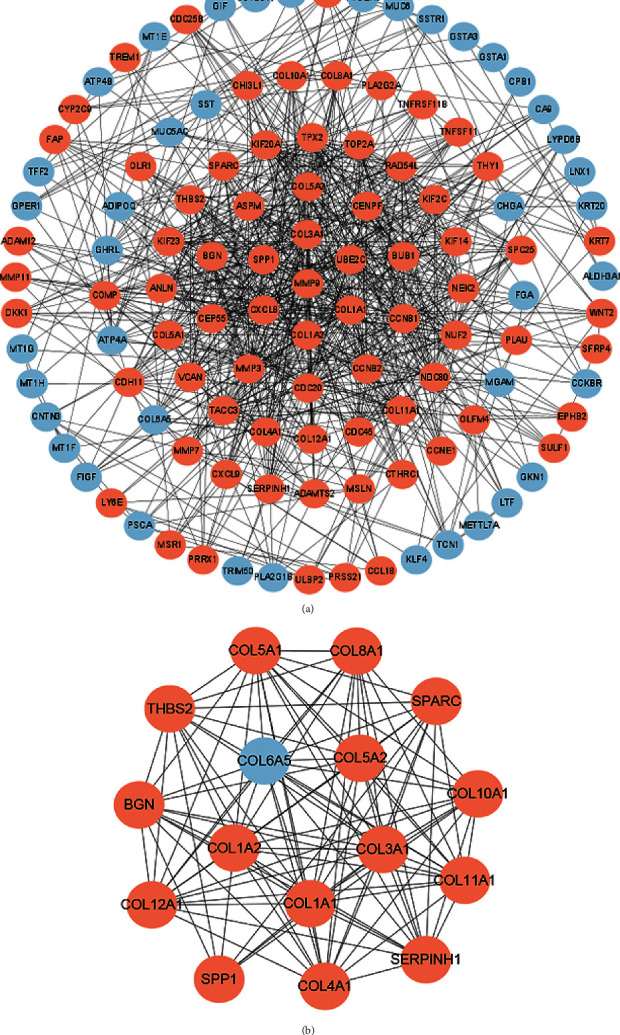
Protein-protein interaction network constructed with overlapping 356 DEGs. (a) PPI network is composed of 117 nodes. (b) The most closely connected module was constructed via MCODE. The black line represents the interaction relationship. The higher the degree, the darker the node.

We analyzed the PPI network of overlapping DEGs to identify key genes and their interactions in the progression of gastric cancer. There were 117 nodes in the PPI network, and we excluded the unconnected nodes. The nodes' degrees were calculated to identify candidate central nodes (Figure 2(a)). Then, we used the MCODE plug-in to select the most closely connected module from the constructed PPI network for further functional analysis. The results showed that the most compact module in the cluster contained 16 genes, namely, COL10A1, COL6A5, SERPINH1, COL5A2, THBS2, COL5A1, BGN, COL4A1, COL3A1, COL11A1, COL12A1, SPARC, COL1A2, SPP1, COL8A1, and COL1A1 (Figure 2(b)).

### 3.4. Survival Analysis

**Figure 3:**
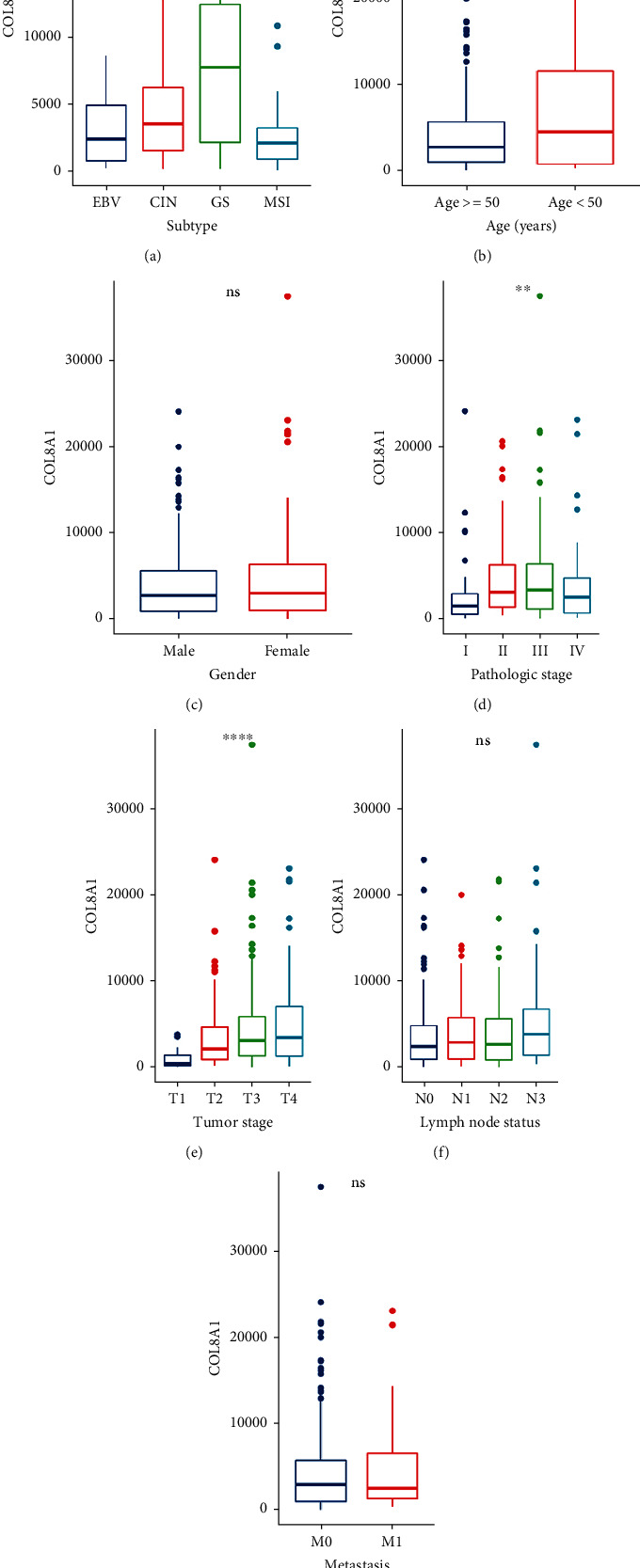
Clinical pathological characteristics of included GC patients.

We performed Kaplan-Meyer (KM) curve analysis on the above 16 genes to identify genes with prognostic significance, and 9 genes with prognostic significance were obtained (Table 2). According to the expression of these 9 genes, we found that COL10A1, SPP1, and COL8A1 showed abundant variation (Table 2). In addition, we found that there was no large-scale cohort study to prove the prognostic value of COL8A1 in STAD patients. Therefore, we chose COL8A1 for further verification. We used the clinical information downloaded from TCGA to analyze the correlation between the expression of COL8A1 and the clinicopathological characteristics and molecular subtype characteristics of STAD patients. Our results show that the genome-stable (GS) subtype has the highest correlation with COL8A1 expression (Figure 3(a)). Analysis of clinicopathological characteristics indicated that the expression of COL8A1 was associated with pathological grade and tumor stage, but not to age, gender, or lymph node status (Figures 3(b)–3(g)).

### 3.5. COL8A1 Was Highly Expressed in STAD and Associated with Poor Prognosis

**Figure 4:**
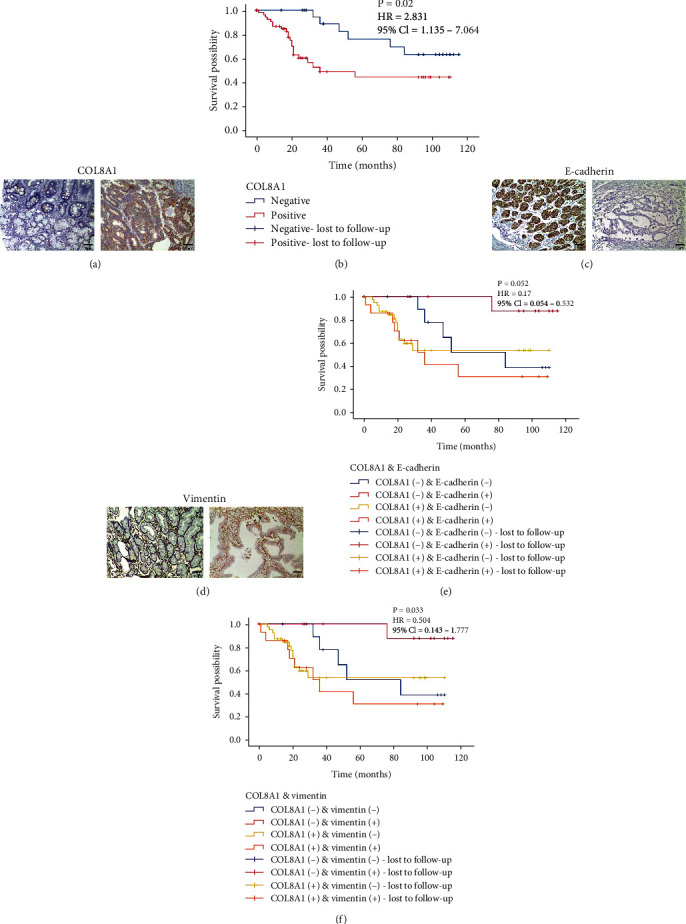
Validation in collected clinical samples. (a) COL8A1 was negatively expressed in normal gastric tissues and positively expressed in gastric adenocarcinoma tissues. (b) The results of survival analysis showed that the high expression of COL8A1 was related to the poor prognosis of GC patients. HR: hazard ratio; 95% CI: 95% confidence interval. (c) E-cadherin was positively expressed in normal gastric tissues and negatively expressed in gastric adenocarcinoma tissues. (d) Vimentin was negatively expressed in normal gastric tissues and positively expressed in gastric adenocarcinoma tissues. (e) The combined prognostic analysis of COL8A1 and E-cadherin. (f) The combined prognostic analysis of COL8A1 and vimentin.

Based on the above analysis, we used our own collected specimens to perform IHC staining of COL8A1 protein to explore the expression of COL8A1 in STAD. As shown in Table 3, COL8A1 was highly expressed in STAD and low in normal gastric tissues (Figure 4(a)).  Table 4 shows the relationship between COL8A1 and the clinical pathological characteristics of the patients. The results indicate that the expression of COL8A1 was related to the tumor's stage and lymph node status. Based on the expression of COL8A1, we divided all patients into a positive expression group and a negative expression group. The results of survival analysis showed that patients with high expression of COL8A1 had poor survival results (Figure 4(b)). These results show that COL8A1 has bearing on the progression of GC and could be used to predict the prognosis.

### 3.6. Gene Set Enrichment Analysis of COL8A1

**Figure 5:**
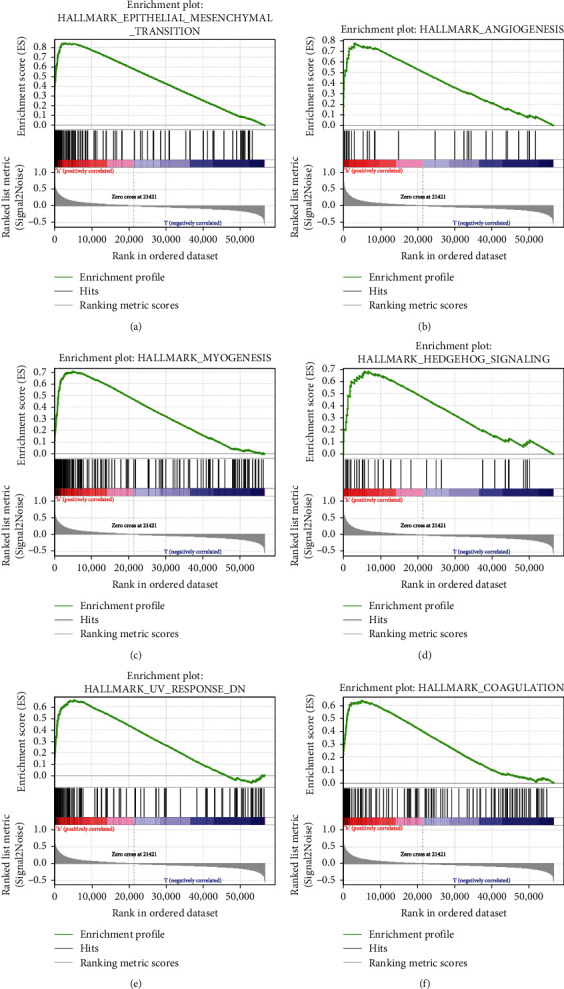
GSEA analysis results of the first six gene sets. The highest point of the curve was the highest point of the enrichment score (ES).

To investigate the carcinogenic mechanism of COL8A1 in GC, we used GSEA to analyze the signal pathways enriched in samples with a high expression of COL8A1. Twenty genomes were identified (FDR < 0.25); the ranked enrichment scores in the top six gene sets are as follows: “epithelial mesenchymal transformation,”, “angiogenesis,” “myogenesis,” “hedgehog_signaling,” “uv response dn (uv response down),” and “coagulation” (Figure 5). These gene sets are all closely related to tumor development [24–26].

### 3.7. The Relationship between COL8A1 and EMT-Related Protein Expression

Based on the results of GSEA analysis, COL8A1 had the highest enrichment score in the EMT pathway. Therefore, we performed IHC staining on EMT-related proteins to explore the relationship between COL8A1 and EMT. The expression of E-cadherin and vimentin proteins is shown in Figures 4(c) and 4(d). The correlation analysis results show that the expression of the E-cadherin protein was negatively correlated with the expression of the COL8A1 protein, and the expression of the vimentin protein was positively correlated with the expression of the COL8A1 protein (Table 5). These results suggest that COL8A1 might promote the progression of STAD through the EMT pathway. The combined prognostic survival analysis showed that COL8A1 and vimentin could be used in combination to predict the prognosis of GC patients (Figures 4(e) and 4(f)). In addition, the expression of E-cadherin and vimentin had no relationship with clinicopathological characteristics of patients (Supplementary Table 1 and Table 2). We also analyzed the effect of E-cadherin protein expression and vimentin protein expression on the survival of patients, but the difference was also not statistically significant (Supplementary Figure 1A-B).

## 4. Discussion

GC is one of the common causes of death of cancer patients in the world. Early diagnosis, timely treatment, and prognosis assessment are of great significance to increase patient survival rates. In our study, a total of 356 differentially expressed genes were obtained through differential gene expression analysis. For exploring the biological functions of these related DEGs, we performed GO and KEGG enrichment analyses. The results showed that DEGs were mainly related to the extracellular matrix components and the physiological functions of the stomach. We identified a module with the most closely connected 16 genes from the PPI network. In order to find biomarkers related to the prognosis of GC, we performed survival analysis on these 16 genes. Finally, we identified COL8A1 as being related to the prognosis of GC for subsequent analysis. GSEA analysis showed that COL8A1 had the highest enrichment score in the EMT pathway. Based on the results of this bioinformatics analysis, we used our own clinically collected gastric cancer samples for immunohistochemical verification to explore the expression of COL8A1 in GC patients and its relationship with the EMT.

COL8A1 is responsible for encoding the *α*1 chain of type VIII collagen and is involved in the formation of the vascular endothelium [27]. Recent research has shown that COL8A1 is dysregulated in a variety of cancers. COL8A1 may affect the progression of colorectal cancer and the prognosis of patients by regulating focal adhesion-related pathways, and the expression of COL8A1 in colorectal cancer is related to the expression of Wnt2 and is linked to the poor survival of patients [28, 29]. COL8A1 may promote breast cancer migration by affecting ECM receptor interactions and cooperation with other genes [30]. There are very few studies of COL8A1 in gastric cancer. Experiments on gastric cancer cells show that the silence of COL8A1 can obviously inhibit cell proliferation, migration, and invasion in GC [31]. We have verified through immunohistochemical analysis that the expression of COL8A1 in GC tissues is distinctly higher than that in normal tissues, and COL8A1 is significantly related to pathological T staging and lymph node metastasis, which suggests that COL8A1 expression may play a part in the progression of gastric cancer. Our survival analysis results showed that COL8A1 could be a biomarker to predict the prognosis of GC.

There are currently 28 types of collagens, which are divided into four families based on the supramolecular structure [32]. These collagen genes all take part in the regulation of EMT in tumors. The downregulation of COL11A1 may affect the migration and invasion cascade of ESCC through the downregulation of EMT [33]. COL6A3 silencing suppresses the expression of MMP-2, MMP-9, and vimentin, then participates in the process of inhibiting EMT of bladder cancer cells [34]. Knocking out COL1A1 can inhibit hepatocellular carcinoma cell migration and invasion by uncontrolled EMT in vitro [35]. The upregulation of COL2A1 may be a biomarker for partial EMT [36]. In bladder cancer, COL1A1 is upregulated. When COL1A1 was knocked down, the EMT process and apoptosis were inhibited [37]. In addition, COL10A1 has a molecular structure similar to COL8A1, and COL10A1 may be an effective costimulator of TGF-*β*1-induced EMT in GC [38]. However, there is currently no study in GC on the relationship between COL8A1 and EMT. Our IHC staining results indicated that COL8A1 was negatively correlated with E-cadherin protein expression and positively correlated with vimentin protein expression. The combined prognostic analysis of COL8A1, E-cadherin, and vimentin indicated that COL8A1 may affect the prognosis of patients through EMT. EMT can promote tumor occurrence, invasion, and metastasis [11]. Many genes can influence the occurrence and development of tumors through EMT and thus affect the prognosis [39–41]. Combining the above collagen family and EMT-related research, our experimental results indicate that COL8A1 might also affect the progression of GC through EMT.

There are some limitations to this study. On the one hand, we had very few STAD tissue samples for IHC experiments, so more clinical tissue samples are needed for validation. Secondly, our exploration of the mechanism by which COL8A1 affected the occurrence and development of GC through EMT is still unclear and needs to be verified by further in vitro and in vivo experiments.

## 5. Conclusions

In summary, this study showed that COL8A1 was upregulated in GC and related to the prognosis of GC patients, indicating that COL8A1could be a biomarker for predicting the prognosis of GC. In addition, COL8A1 may affect the progression of GC through EMT.

## Figures and Tables

**Table 1:** Detailed information of the four GEO datasets.

Dataset	Platform	Number of samples (normal/tumor)
GSE19826	GPL570	24 (12/12)
GSE118916	GPL15207	30 (15/15)
GSE79973	GPL570	20 (10/10)
GSE66229	GPL570	400 (100/300)

**Table 2:** Survival analysis results of 16 genes in the module.

Gene	HR	95% CI	*P* value
COL10A1	1.569	1.099-2.240	0.013
COL6A5	1.058	0.742-1.508	0.756
SERPINH1	1.081	0.758-1.541	0.668
COL5A2	1.607	1.126-2.294	0.009
THBS2	1.273	0.893-1.815	0.182
COL5A1	1.489	1.043-2.125	0.028
BGN	1.246	0.874-1.777	0.224
COL4A1	1.665	1.167-2.375	0.005
COL3A1	1.426	0.999-2.036	0.049
COL11A1	1.245	0.873-1.776	0.225
COL12A1	1.555	1.090-2.218	0.015
SPARC	1.659	1.162-2.370	0.005
COL1A2	1.309	0.917-1.868	0.135
SPP1	1.515	1.060-2.164	0.021
COL8A1	1.637	1.146-2.339	0.006
COL1A1	1.330	0.931-1.900	0.110

**Table 3:** The expression of COL8A1 in STAD and normal gastric tissues.

COL8A1	Total	(-)	(+)	(++)	(+++)	*χ* ^2^	*P* value
STAD	119	35	47	29	8		
Normal	40	23	8	7	2	8.0	0.045

**Table 4:** The relationship between the expression of COL8A1, E-cadherin, and vimentin in STAD.

		Total	COL8A1 (-)	COL8A1 (+)	COL8A1 (++)	COL8A1 (+++)	*r*	*P* value
E-cadherin	(-) (+)	81 38	17 18	36 11	23 6	7 1	-0.275	0.002
Vimentin	(-) (+)	51 68	22 13	20 27	8 21	1 7	0.309	0.001

**Table 5:** The relationship between the expression of COL8A1 and clinicopathological characteristics.

COL8A1	Total (*N* = 119)	Negative (*N* = 35)	Positive (*N* = 84)	*P* value
*Age (years)*				0.905
Age < 50	23 (19.3%)	7 (20.0%)	16 (19.0%)	
Age ≥ 50	96 (80.7%)	28 (80.0%)	68 (81.0%)	
*Gender*				0.692
Female	18 (15.1%)	6 (17.1%)	12 (14.3%)	
Male	101 (84.9%)	29 (82.9%)	72 (85.7%)	
*Differentiation degree*				0.922
Poorly	37 (31.1%)	11 (31.4%)	26 (31.0%)	
Moderately	78 (65.5%)	23 (65.7%)	55 (65.5%)	
Well	4 (3.4%)	1 (2.9%)	3 (3.6%)	
*Pathologic stage*				
I	8 (6.7%)	4 (11.4%)	4 (4.8%)	0.143
II	29 (24.4%)	10 (28.6%)	19 (22.6%)	
III	64 (53.8%)	17 (48.6%)	47 (56.0%)	
IV	18 (15.1%)	4 (11.4%)	14 (16.7%)	
*T stage*				
T1	1 (0.8%)	1 (2.9%)	0 (0%)	0.035
T2	13 (10.9%)	6 (17.1%)	7 (8.3%)	
T3	49 (41.2%)	16 (45.7%)	33 (39.3%)	
T4	56 (47.1%)	12 (34.3%)	44 (52.4%)	
*Lymph node status*				
N0	29 (24.4%)	10 (28.6%)	19 (22.6%)	0.030
N1	24 (20.2%)	11 (31.4%)	13 (15.5%)	
N2	36 (30.3%)	11 (31.4%)	25 (29.8%)	
N3	30 (25.2%)	3 (8.6%)	27 (32.1%)	
*Metastasis*				0.565
M0	102 (85.7%)	31 (88.6%)	71 (84.5%)	
M1	17 (14.3%)	4 (11.4%)	13 (15.5%)	

## Data Availability

The authors confirm that the data supporting the findings of this study are available within the article.

## Abbreviations

GC: Gastric cancer
OS: Overall survival
STAD: Stomach adenocarcinoma
COL8A1: Collagen type VIII alpha-1 chain
EMT: Epithelial-mesenchymal transition
GEO: Gene Expression Omnibus
TCGA: The Cancer Genome Atlas
DEGs: Differential genes
GO: Gene Ontology
KEGG: Kyoto Encyclopedia of Genes and Genomes
PPI: Protein-protein interaction
GSEA: Gene set enrichment analysis
CC: Cellular components
BP: Biological processes
MF: Molecular functions
FFPE: Formalin-fixed and paraffin-embedded
H&E: Hematoxylin and eosin
AJCC: The American Joint Committee on Cancer
TNM: Tumor, lymph node, and metastasis
IHC: Immunohistochemistry
EDTA: Ethylenediaminetetraacetic acid.

## Supplementary Materials

Supplementary Materials Supplementary Figure 1: (A) survival analysis result of E-cadherin using collected clinical sample information. (B) Survival analysis result of vimentin using collected clinical sample information. HR was the hazard ratio, and 95% CI was the 95% confidence interval. Supplementary Table 1: the relationship between the expression of E-cadherin and clinicopathological characteristics. Supplementary Table 2: the relationship between the expression of vimentin and clinicopathological characteristics.

## References

[1] Smyth E. C., Nilsson M., Grabsch H. I., van Grieken N. C., Lordick F. (2020). Gastric cancer. *Lancet*.

[2] Wu D., Zhang P., Ma J. (2019). Serum biomarker panels for the diagnosis of gastric cancer. *Cancer Medicine*.

[3] Alsina M., Diez M., Tabernero J. (2021). Emerging biological drugs for the treatment of gastroesophageal adenocarcinoma. *Expert Opinion on Emerging Drugs*.

[4] Asplund J., Kauppila J. H., Mattsson F., Lagergren J. (2018). Survival trends in gastric adenocarcinoma: a population-based study in Sweden. *Annals of Surgical Oncology*.

[5] Shuttleworth C. A. (1997). Type VIII collagen. *The International Journal of Biochemistry & Cell Biology*.

[6] Paulus W., Sage E. H., Liszka U., Iruela-Arispe M. L., Jellinger K. (1991). Increased levels of type VIII collagen in human brain tumours compared to normal brain tissue and non-neoplastic cerebral disorders. *British Journal of Cancer*.

[7] Zhao Y., Jia L., Mao X., Xu H., Wang B., Liu Y. (2009). siRNA-targeted COL8A1 inhibits proliferation, reduces invasion and enhances sensitivity to D-limonence treatment in hepatocarcinoma cells. *IUBMB Life*.

[8] Willumsen N., Jorgensen L. N., Karsdal M. A. (2019). Vastatin (the NC1 domain of human type VIII collagen a1 chain) is linked to stromal reactivity and elevated in serum from patients with colorectal cancer. *Cancer Biology & Therapy*.

[9] Hanahan D., Weinberg R. A. (2011). Hallmarks of cancer: the next generation. *Cell*.

[10] Peng Z., Wang C. X., Fang E. H., Wang G. B., Tong Q. (2014). Role of epithelial-mesenchymal transition in gastric cancer initiation and progression. *World Journal of Gastroenterology*.

[11] Dongre A., Weinberg R. A. (2019). New insights into the mechanisms of epithelial-mesenchymal transition and implications for cancer. *Nature Reviews Molecular Cell Biology*.

[12] Yeung K. T., Yang J. (2017). Epithelial–mesenchymal transition in tumor metastasis. *Molecular Oncology*.

[13] Bure I. V., Nemtsova M. V., Zaletaev D. V. (2019). Roles of E-cadherin and noncoding RNAs in the epithelial–mesenchymal transition and progression in gastric cancer. *International Journal of Molecular Sciences*.

[14] Can T. (2014). Introduction to bioinformatics. *Methods in Molecular Biology*.

[15] Wu J., Zhou J., Xu Q. (2021). Identification of key genes driving tumor associated macrophage migration and polarization based on immune fingerprints of lung adenocarcinoma. *Frontiers in Cell and Development Biology*.

[16] Wu H., Tian W., Tai X. (2021). Identification and functional analysis of novel oncogene DDX60L in pancreatic ductal adenocarcinoma. *BMC Genomics*.

[17] Zou Q., Lv Y., Gan Z., Liao S., Liang Z. (2021). Identification and validation of a malignant cell subset marker-based polygenic risk score in stomach adenocarcinoma through integrated analysis of bulk and single-cell RNA sequencing data. *Frontiers in Cell and Development Biology*.

[18] Ritchie M. E., Phipson B., Wu D. (2015). Limma powers differential expression analyses for RNA-sequencing and microarray studies. *Nucleic Acids Research*.

[19] Kolde R., Laur S., Adler P., Vilo J. (2012). Robust rank aggregation for gene list integration and meta-analysis. *Bioinformatics*.

[20] Ding K., Qiu W., Yu D. (2021). Bioinformatics analysis of ZBTB16 as a prognostic marker for Ewing’s sarcoma. *BioMed Research International*.

[21] Raglow Z., Thomas S. M. (2015). Tumor matrix protein collagen XI*α*1 in cancer. *Cancer Letters*.

[22] Xie J., Pang Y., Wu X. (2021). Taxifolin suppresses the malignant progression of gastric cancer by regulating the AhR/CYP1A1 signaling pathway. *International Journal of Molecular Medicine*.

[23] Wang Z., Wang Z., Hu X., Han Q., Chen K., Pang G. (2021). Extracellular matrix-associated pathways promote the progression of gastric cancer by impacting the dendritic cell axis. *International Journal of General Medicine*.

[24] Folkman J. (1990). What is the evidence that tumors are angiogenesis dependent?. *Journal of the National Cancer Institute*.

[25] Shen Y., Chan G., Xie M., Zeng W., Liu L. (2019). Identification of master regulator genes of UV response and their implications for skin carcinogenesis. *Carcinogenesis*.

[26] González-Mariscal L., Miranda J., Gallego-Gutiérrez H., Cano-Cortina M., Amaya E. (2020). Relationship between apical junction proteins, gene expression and cancer. *Biochimica et Biophysica Acta, Biomembranes*.

[27] Greenhill N. S., Rüger B. M., Hasan Q., Davis P. F. (2000). The *α*1(VIII) and *α*2(VIII) collagen chains form two distinct homotrimeric proteins in vivo. *Matrix Biology*.

[28] Zhang L., Jiang X., Li Y. (2020). Clinical correlation of Wnt2 and COL8A1 with colon adenocarcinoma prognosis. *Frontiers in Oncology*.

[29] Shang J., Wang F., Chen P. (2018). Co-expression network analysis identified COL8A1 is associated with the progression and prognosis in human colon adenocarcinoma. *Digestive Diseases and Sciences*.

[30] Peng W., Li J. D., Zeng J. J. (2020). Clinical value and potential mechanisms of COL8A1 upregulation in breast cancer: a comprehensive analysis. *Cancer Cell International*.

[31] Zhou J., Song Y., Gan W. (2020). Upregulation of COL8A1 indicates poor prognosis across human cancer types and promotes the proliferation of gastric cancer cells. *Oncology Letters*.

[32] Xu S., Xu H., Wang W. (2019). The role of collagen in cancer: from bench to bedside. *Journal of Translational Medicine*.

[33] Zhang B., Zhang C., Yang X. (2018). Cytoplasmic collagen XI*α*I as a prognostic biomarker in esophageal squamous cell carcinoma. *Cancer Biology & Therapy*.

[34] Huang Y., Li G., Wang K. (2018). Collagen type VI alpha 3 chain promotes epithelial-mesenchymal transition in bladder cancer cells via transforming growth factor *β* (TGF-*β*)/Smad pathway. *Medical Science Monitor*.

[35] Ma H. P., Chang H. L., Bamodu O. A. (2019). Collagen 1A1 (COL1A1) is a reliable biomarker and putative therapeutic target for hepatocellular carcinogenesis and metastasis. *Cancers*.

[36] Karaosmanoğlu O., Banerjee S., Sivas H. (2018). Identification of biomarkers associated with partial epithelial to mesenchymal transition in the secretome of slug over-expressing hepatocellular carcinoma cells. *Cellular Oncology (Dordrecht)*.

[37] Zhu H., Chen H., Wang J., Zhou L., Liu S. (2019). Collagen stiffness promoted non-muscle-invasive bladder cancer progression to muscle-invasive bladder cancer. *Oncotargets and Therapy*.

[38] Li T., Huang H., Shi G. (2018). TGF-*β*1-SOX9 axis-inducible COL10A1 promotes invasion and metastasis in gastric cancer via epithelial-to-mesenchymal transition. *Cell Death & Disease*.

[39] Wu G., Yang Y., Zhu Y. (2021). Comprehensive analysis to identify the epithelial-mesenchymal transition-related immune signatures as a prognostic and therapeutic biomarkers in hepatocellular carcinoma. *Frontiers in Surgery*.

[40] Ye L., Wang X., Li B. (2021). Expression profile of epithelial-mesenchymal transition-related genes as a prognostic biomarker for endometrial cancer. *Journal of Cancer*.

[41] Garinet S., Didelot A., Denize T. (2021). Clinical assessment of the miR-34, miR-200, ZEB1 and SNAIL EMT regulation hub underlines the differential prognostic value of EMT miRs to drive mesenchymal transition and prognosis in resected NSCLC. *British Journal of Cancer*.

