# Supplementary material for: COL8A1 Predicts the Clinical Prognosis of Gastric Cancer and Is Related to Epithelial-Mesenchymal Transition
Source: Biomed Res Int. 2022 Jun 21;2022:7567447. doi: 10.1155/2022/7567447 (PMC9239809; doi:10.1155/2022/7567447)
Supplement: Supplementary Materials — Supplementary Figure 1: (A) survival analysis result of E-cadherin using collected clinical sample information. (B) Survival analysis result of vimentin using collected clinical sample information. HR was the hazard ratio, and 95% CI was the 95% confidence interval. Supplementary Table 1: the relationship between the expression of E-cadherin and clinicopathological characteristics. Supplementary Table 2: the relationship between the expression of vimentin and clinicopathological characteristics. [file 7567447.f1.zip › Supplementary Table.docx]

**Supplementary Table 1 The relationship between the expression of E-cadherin and clinicopathological characteristics**

| **E-cadherin** | **Total (N=119)** | **Negative (N=83)** | **Positive (N=36)** | ***P* value** |
| --- | --- | --- | --- | --- |
| **Age (years)** |  |  |  | 0.983 |
| age < 50 | 23 (19.3%) | 16 (19.3%) | 7 (19.4%) |  |
| age >= 50 | 96 (80.7%) | 67 (80.7%) | 29 (80.6%) |  |
| **Gender** |  |  |  | 0.804 |
| female | 18 (15.1%) | 13 (15.7%) | 5 (13.9%) |  |
| male | 101 (84.9%) | 70 (84.3%) | 31 (86.1%) |  |
| **Differentiation degree** |  |  |  | 0.732 |
| poorly | 37 (31.1%) | 24 (28.9%) | 13 (36.1%) |  |
| moderately | 78 (65.5%) | 56 (67.5%) | 22 (61.1%) |  |
| well | 4 (3.4%) | 3 (3.6%) | 1 (2.8%) |  |
| **Pathologic stage** |  |  |  |  |
| I | 8 (6.7%) | 6 (7.2%) | 2 (5.6%) | 0.838 |
| II | 29 (24.4%) | 21 (25.3%) | 8 (22.2%) |  |
| III | 64 (53.8%) | 45 (54.2%) | 19 (52.8%) |  |
| IV | 18 (15.1%) | 11 (13.3%) | 7 (19.4%) |  |
| **T Stage** |  |  |  | 0.816 |
| T1 | 1 (0.8%) | 0 (0%) | 1 (2.8%) |  |
| T2 | 13 (10.9%) | 11 (13.3%) | 2 (5.6%) |  |
| T3 | 49 (41.2%) | 33 (39.8%) | 16 (44.4%) |  |
| T4 | 56 (47.1%) | 39 (47.0%) | 17 (47.2%) |  |
| **Lymph node status** |  |  |  |  |
| N0 | 29 (24.4%) | 20 (24.1%) | 9 (25.0%) | 0.114 |
| N1 | 24 (20.2%) | 15 (18.1%) | 9 (25.0%) |  |
| N2 | 36 (30.3%) | 22 (26.5%) | 14 (38.9%) |  |
| N3 | 30 (25.2%) | 26 (31.3%) | 4 (11.1%) |  |
| **Metastasis** |  |  |  |  |
| M0 | 102 (85.7%) | 73 (88.0%) | 29 (80.6%) | 0.290 |
| M1 | 17 (14.3%) | 10 (12.0%) | 7 (19.4%) |  |

**Supplementary Table 2 The relationship between the expression of Vimentin and clinicopathological characteristics**

| **Vimentin** | **Total (N=119)** | **Negative (N=51)** | **Positive (N=68)** | ***P* value** |
| --- | --- | --- | --- | --- |
| **Age (years)** |  |  |  | 0.592 |
| age < 50 | 23 (19.3%) | 11 (21.6%) | 12 (17.6%) |  |
| age >= 50 | 96 (80.7%) | 40 (78.4%) | 56 (82.4%) |  |
| **Gender** |  |  |  | 0.089 |
| female | 18 (15.1%) | 11 (21.6%) | 7 (10.3%) |  |
| male | 101 (84.9%) | 40 (78.4%) | 61 (89.7%) |  |
| **Differentiation degree** |  |  |  | 0.954 |
| moderately | 78 (65.5%) | 33 (64.7%) | 45 (66.2%) |  |
| poorly | 37 (31.1%) | 16 (31.4%) | 21 (30.9%) |  |
| well | 4 (3.4%) | 2 (3.9%) | 2 (2.9%) |  |
| **Pathologic stage** |  |  |  | 0.253 |
| I | 8 (6.7%) | 3 (5.9%) | 5 (7.4%) |  |
| II | 29 (24.4%) | 13 (25.5%) | 16 (23.5%) |  |
| III | 64 (53.8%) | 31 (60.8%) | 33 (48.5%) |  |
| IV | 18 (15.1%) | 4 (7.8%) | 14 (20.6%) |  |
| **T Stage** |  |  |  | 0.550 |
| T1 | 1 (0.8%) | 1 (2.0%) | 0 (0%) |  |
| T2 | 13 (10.9%) | 5 (9.8%) | 8 (11.8%) |  |
| T3 | 49 (41.2%) | 19 (37.3%) | 30 (44.1%) |  |
| T4 | 56 (47.1%) | 26 (51.0%) | 30 (44.1%) |  |
| **Lymph node status** |  |  |  | 0.661 |
| N0 | 29 (24.4%) | 11 (21.6%) | 18 (26.5%) |  |
| N1 | 24 (20.2%) | 11 (21.6%) | 13 (19.1%) |  |
| N2 | 36 (30.3%) | 18 (35.3%) | 18 (26.5%) |  |
| N3 | 30 (25.2%) | 11 (21.6%) | 19 (27.9%) |  |
| **Metastasis** |  |  |  | 0.082 |
| M0 | 102 (85.7%) | 47 (92.2%) | 55 (80.9%) |  |
| M1 | 17 (14.3%) | 4 (7.8%) | 13 (19.1%) |  |
