# Supplementary figures and images for: COL8A1 Predicts the Clinical Prognosis of Gastric Cancer and Is Related to Epithelial-Mesenchymal Transition
Source: Biomed Res Int. 2022 Jun 21;2022:7567447. doi: 10.1155/2022/7567447 (PMC9239809; doi:10.1155/2022/7567447)

**A**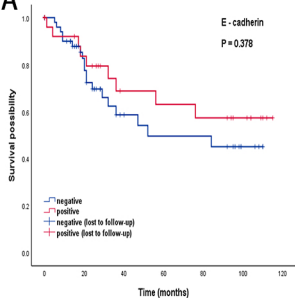**B**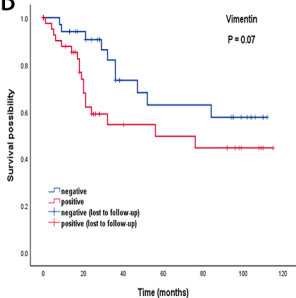

Supplement: Supplementary Materials — Supplementary Figure 1: (A) survival analysis result of E-cadherin using collected clinical sample information. (B) Survival analysis result of vimentin using collected clinical sample information. HR was the hazard ratio, and 95% CI was the 95% confidence interval. Supplementary Table 1: the relationship between the expression of E-cadherin and clinicopathological characteristics. Supplementary Table 2: the relationship between the expression of vimentin and clinicopathological characteristics. [file 7567447.f1.zip › Supplementary Figure 1.pdf]
